# Management of hyperkalemia in chronic heart failure using sodium zirconium cyclosilicate

**DOI:** 10.1002/clc.23683

**Published:** 2021-07-15

**Authors:** Akira Oshima, Teruhiko Imamura, Nikhil Narang, Koichiro Kinugawa

**Affiliations:** ^1^ Second Department of Internal Medicine University of Toyama Toyama Japan; ^2^ Advocate Christ Medical Center Oak Lawn Illinois USA

**Keywords:** chronic kidney disease, hyperkalemia, hypokalemia, potassium

## Abstract

**Background:**

Sodium zirconium cyclosilicate (SZC), a newly‐developed selective potassium binder, has been clinically available to treat hyperkalemia. SZC might be a promising option to manage hyperkalemia, particularly in patients with heart failure, who often require potassium‐sparing medications. However, the optimal initial dose of SZC therapy at a loading dose (30 g per day for the initial 2 days) versus a maintenance dose (5 g per day) remains unknown.

**Methods:**

Consecutive patients with heart failure and hyperkalemia who received 2‐day SZC therapy were retrospectively included. Safety and efficacy of SZC therapy were compared between the two strategies (maintenance versus loading).

**Results:**

We had 16 patients (76 years old, 11 men) who received 2‐day SZC therapy (4 maintenance dose group and 12 loading dose group). Serum potassium decreased 0.7 mEqL/L by 2‐day maintenance dose therapy and 1.3 mEq/L by 2‐day loading dose therapy. Following 2‐day SZC therapy, 25% of patients had hypokalemia, which was defined as serum potassium <4.0 mEq/L. Baseline lower serum potassium level was associated with the post‐SZC hypokalemia.

**Conclusions:**

SZC immediately decreases approximately 1.0 mEq/L of serum potassium in patients with heart failure and hyperkalemia. However, caution should be exercised when utilizing SZC at a loading dose specifically in those with mild hyperkalemia to prevent iatrogenic hypokalemia.

## 1 INTRODUCTION

Hyperkalemia (generally defined as serum potassium level > 5.0 mEq/L) is often present in chronic heart failure due to concomitant chronic kidney disease. Its presence often limits up‐titration of guideline‐directed medical therapies and indirectly may affect long‐term clinical risk.^1^, ^2^

Recently, sodium zirconium cyclosilicate (SZC), a non‐absorbed, non‐polymer zirconium silicate compound that exchanges hydrogen and sodium for potassium and ammonium ions in the gastrointestinal tract, has been clinically available to treat hyperkalemia.^3^ A loading dose with 30 g per day of SZC is generally recommended as initial 2‐day therapy. If post‐treatment hypokalemia that sometimes triggers cardiac arrhythmia is worried, a maintenance dose with 5 g per day of SZC might be preferred.^4^

Appropriate usage of SZC would be essential for the management of hyperkalemia and successful administration and up‐titration of renin‐angiotensin aldosterone inhibitors and mineralocorticoid receptor antagonists, preventing fatal abnormality in the serum potassium level. In this study, we investigated (1) the actual degree of reduction in serum potassium levels following the 2‐day SZC therapy with both loading and maintenance dose in a representative heart failure cohort (efficacy analysis) and (2) factors associating with hypokalemia following the 2‐day SZC therapy (safety analysis).

## 2 METHODS

### 2.1 Patient selection

Consecutive patients with heart failure and hyperkalemia, who received SZC at least for 2 days during the index hospitalization between July 2020 and November 2020, were included in this retrospective study. Definition of heart failure was based on the Framingham criteria. Patients dependent on hemodiafiltration and those receiving sodium polystyrene sulphonate did not receive SZC and were excluded from this study. No patients had mineralocorticoid receptor antagonists given the existence of hyperkalemia.

Serum potassium levels were measured at baseline (day 0) and 2 days later (day 2). Informed consent was obtained from all participants and this study was approved by the institutional ethical board beforehand.

### 2.2 SZC therapy

In patients with hyperkalemia, SZC was initiated at a loading dose (30 g per day) or as a maintenance dose (5 g per day) for 2 days at the attending physicians' discretion. All other medical therapies were continued without adjustment.

### 2.3 Data collection

Baseline demographic, laboratory, and medication data obtained within 24 h prior to the SZC administration were collected. Of note, baseline (day 0) and day 2 serum potassium levels were obtained. The change in serum potassium level during the two‐day SZC therapy was calculated as a primary endpoint.

### 2.4 Statistical analyses

Statistical analyses were performed using the SPSS Statistics 22 (SPSS Inc, Armonk, IL). Two‐sided p‐values <0.05 were considered statistically significant. All variables were assumed as non‐parametric data given a small sample size. Changes in serum potassium levels during the two‐day SZC therapy were compared between the loading dose group and maintenance dose group using a Mann–Whitney U test as a primary outcome (efficacy analysis). Baseline characteristics were compared between those who suffered hypokalemia (defined as serum potassium level < 4.0 mEq/L) on day 2 and those without hypokalemia on day 2 (safety analysis).

## 3 RESULTS

### 3.1 Baseline characteristics

In total, 16 heart failure patients with hyperkalemia (76 [67, 86] years old, 11 males) received two‐day SZC therapy. Among them, 5 received SZC at a maintenance dose and the remaining 11 patients at a loading dose. Their baseline serum potassium level was 5.5 (5.3, 5.8) mEq/L. No patients received mineralocorticoid receptor antagonists. All patients received potassium‐restriction diets (<2000 mg daily of potassium intake). No patients had anorexia. During the 2‐day SZC therapy, no patients had peripheral edema, fatigue, nasopharyngitis, and upper respiratory tract infection.

### 3.2 Changes in serum potassium levels (efficacy analysis)

**FIGURE 1:**
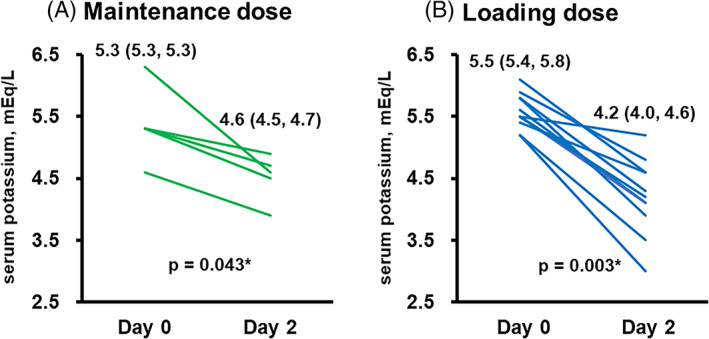
Trends of serum potassium level during the 2‐day SZC therapy in the maintenance dose group (A) and in the loading dose group (B). Data were stated as median and interquartile. Trends were assessed using a Wilcoxon signed‐rank test. *p < 0.05

**FIGURE 2:**
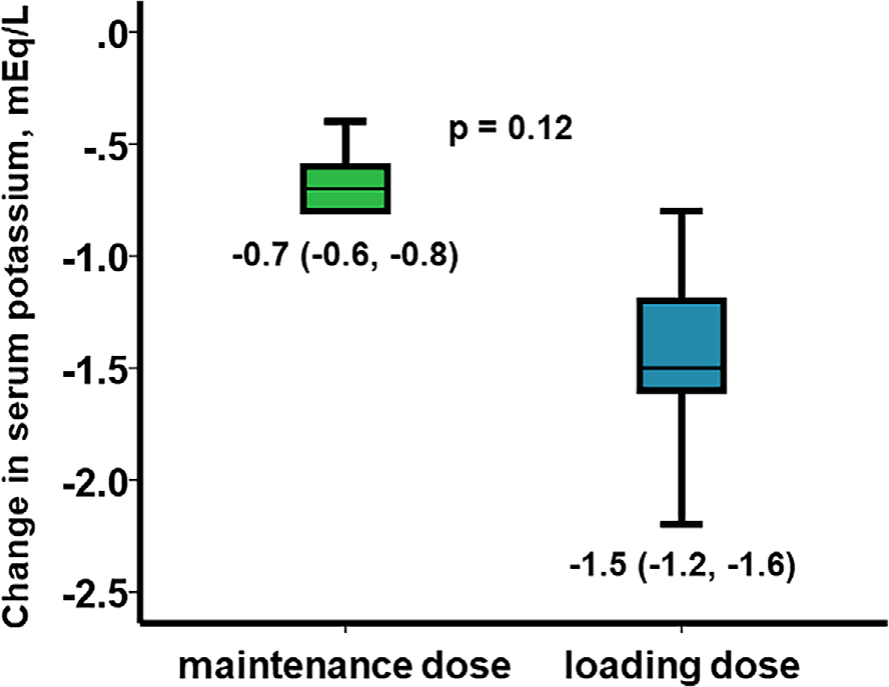
Changes in serum potassium level were compared between the two groups. Data were stated as median and interquartile. Changes were compared using a Mann–Whitney U test. *p < 0.05

During the 2‐day SZC therapy, serum potassium level decreased from 5.3 (5.3, 5.3) mEq to 4.6 (4.5, 4.7) mEq in the maintenance dose group (Figure 1(A)), whereas serum potassium level decreased from 5.5 (5.4, 5.8) to 4.2 (4.0, 4.6) mEq/L in the loading dose group (Figure 1(B)). Changes in serum potassium level were −0.7 (−0.6, −0.8) mEq/L in the maintenance dose group and −1.5 (−1.2, −1.6) mEq/L in the loading dose group (p = 0.12; Figure 2).

### 3.3 Factors associating with hypokalemia following SZC therapy (safety analysis)

Four out of 16 patients suffered hypokalemia following the 2‐day SZC therapy. Of them, two patients had non‐sustained ventricular tachycardia.

The patients who suffered hypokalemia had significantly lower baseline serum potassium level (5.2 versus 5.5 mEq/L, p = 0.048) and a trend toward higher age (86 versus 74 years, p = 0.10), lower estimated glomerular filtration ratio (23.9 versus 37.2 ml/min/1.73m^2^, p = 0.21), and higher prevalence of diuretics (75% versus 33%, p = 0.19) (Table 1). The prevalence of SZC loading did not significantly differ between the two groups (75% in the hypokalemia group versus 67% in the normokalemia group, p = 0.64).

**TABLE 1:** Baseline characteristics stratified by the serum potassium levels on day 2 following the initiation of SZC

	Total (*N* = 16)	S‐K < 4.0 mEq/L (*N* = 4)	S‐K ≥ 4.0 mEq/L (*N* = 12)	p value
Age, years	76 (67, 86)	86 (72, 90)	74 (66, 79)	0.10
Male sex	11 (69%)	3 (75%)	8 (67%)	0.64
Body mass index	20.2 (17.1, 22.1)	21.1 (17.9, 25.9)	19.4 (17.1, 22.1)	0.52
Ischemic etiology	5 (31%)	2 (50%)	3 (25%)	0.37
Atrial fibrillation	3 (19%)	1 (25%)	2 (17%)	0.61
Diabetes mellitus	4 (25%)	2 (50%)	2 (17%)	0.25
Systolic blood pressure, mmHg	121 (101, 131)	129 (108, 144)	115 (99, 130)	0.26
Diastolic blood pressure, mmHg	64 (55, 74)	59 (48, 79)	66 (55, 74)	0.45
Heart rate, bpm	75 (65, 92)	81 (74, 95)	71 (61, 92)	0.45
Estimated glomerular filtration ratio, ml/min/1.73m^2^	34.0 (21.3, 51.9)	23.9 (15.7, 40.4)	37.2 (25.4, 59.3)	0.21
Plasma B‐type natriuretic peptide, pg/ml	190 (66, 296)	128 (69, 329)	220 (65, 291)	0.86
Beta blocker	8 (50%)	2 (50%)	6 (50%)	0.83
Renin angiotensin aldosterone system inhibitor	8 (50%)	3 (75%)	5 (29%)	0.29
Diuretics	7 (44%)	3 (75%)	4 (33%)	0.19
Baseline S‐K, mEq/L	5.5 (5.3, 5.8)	5.2 (4.8, 5.7)	5.5 (5.3, 5.9)	0.048^a^
Two‐day loading dose (30 g per day)	11 (69%)	3 (75%)	8 (67%)	0.64

*Note:* S‐K, serum potassium. Continuous variables were expressed as median and interquartile (25% and 75%) and compared using the Mann–Whitney U test. Categorical variables were expressed as number and percentage and compared using Fisher's exact test.

^a^ p < 0.05.

## 4 DISCUSSION

In this study, we investigated the change in serum potassium level following 2‐day SZC therapy in patients with chronic heart failure and hyperkalemia. Our main findings were: (1) Serum potassium decreased approximately 0.7 mEqL/L by 2‐day maintenance dose therapy (5 g per day) and approximately 1.3 mEq/L by 2‐day loading dose therapy (30 g per day) (efficacy analysis); (2) 25% of patients suffered hypokalemia (defined as serum potassium <4.0 mEq/L) following 2‐day SZC therapy. A baseline lower serum potassium level was associated with the development of post‐SZC hypokalemia (safety analysis).

### 4.1 Efficacy of SZC

The HARMONIZE phase III double‐blind randomized control study demonstrated that a 2‐day SZC therapy at a loading dose decreased 1.28 mEq/L of serum potassium level.^3^ This study included only 19% of heart failure patients. The efficacy of SZC might differ in heart failure populations due to the presence of therapies which directly modulate potassium absorption and excretion.^1^ In this study of patients with heart failure, we confirmed that the average decrease in serum potassium was similar to the HARMONIZE results (−1.3 mEq/L in the loading group). We notably observed a reduced efficacy of the maintenance dose compared to the loading dose. The finding should be useful to determine which strategy (loading or maintenance) to choose as an initial dose considering the target serum potassium level.

### 4.2 Safety of SZC to avoid hypokalemia

The development of hypokalemia following the SZC initiation remains an apparent risk that requires close monitoring.^3^ Established serum targets for potassium levels are >4.0 mEq/L in patients with heart failure to reduce the risk of cardiac arrhythmias.^5^ This is a rationale why we defined serum potassium <4.0 mEq/L as ''hypokalemia'' in this study.

Relatively lower baseline serum potassium level (between 5.0 and 5.5 mEq/L) was associated with post‐SZC hypokalemia. Higher age, impaired renal function, and the use of diuretics were collectively associated with the development of hypokalemia. Elderly heart failure patients often have chronic kidney disease, which require diuretics therapy for the management of systemic congestion. The use of diuretics worsens renal function via vascular hypovolemia, impaired pre‐renal blood flow, and activation of renin‐angiotensin‐aldosterone system. Diuretics is in general increases the secretion of potassium in urine and reduces serum potassium. Therefore, it is not surprising that these characteristics are associated with extreme reduction in serum potassium level during the SZC therapy. Caution should be exercised when initiating loading doses of SZC particularly for these specific cohort. Of note, the cohort with these characteristics is just the dominant target of SZC therapy.

### 4.3 Limitations and future directions

This study is a proof of concept and consisted of a small sample size. We believe these findings are relevant nevertheless in regards to the risk of loading therapy in patients with heart failure and mild hyperkalemia (serum potassium between 5.0 and 5.5 mEq/L). For those with risks of post‐SZC hypokalemia, we recommend initiating SZC at a maintenance dose, instead of a loading dose, particularly when hyperkalemia is mild.

We acknowledge the presence of confounding variables in this study which may be better mitigated in a larger prospective analysis. Our findings should be validated in a larger‐scale cohort including comprehensive clinical data. We observed only 2‐day SZC therapy, and a long‐term study should be conducted. Our patients received potassium‐restricting diet and none of them had anorexia. Our findings might not simply be adopted in such cohort.

## 5 CONCLUSION

SZC is a promising therapeutic option that immediately decreases approximately 1.0 mEq/L of serum potassium in patients with heart failure and hyperkalemia. However, caution should be exercised when utilizing SZC at a loading dose specifically in those with mild hyperkalemia. A maintenance dose, instead of loading dose, would be recommended in such a cohort as an initial dose.

## CONFLICT OF INTEREST

The authors declare no potential conflict of interest.

## Data Availability

Data are available from corresponding author upon reasonable request.
